# MSA-MOT: Multi-Stage Association for 3D Multimodality Multi-Object Tracking

**DOI:** 10.3390/s22228650

**Published:** 2022-11-09

**Authors:** Ziming Zhu, Jiahao Nie, Han Wu, Zhiwei He, Mingyu Gao

**Affiliations:** 1 The School of Electronic Information, Hangzhou Dianzi University, Hangzhou 310018, China; 2 Zhejiang Province Key Laboratory of Equipment Electronics, Hangzhou 310018, China

**Keywords:** 3D multi-object tracking, multimodal, multi-stage association, track management

## Abstract

Three-dimensional multimodality multi-object tracking has attracted great attention due to the use of complementary information. However, such a framework generally adopts a one-stage association approach, which fails to perform precise matching between detections and tracklets, and, thus, cannot robustly track objects in complex scenes. To address this matching problem caused by one-stage association, we propose a novel multi-stage association method, which consists of a hierarchical matching module and a customized track management module. Specifically, the hierarchical matching module defines the reliability of the objects by associating multimodal detections, and matches detections with trajectories based on the reliability in turn, which increases the utilization of true detections, and, thus, guides accurate association. Then, based on the reliability of the trajectories provided by the matching module, the customized track management module sets maximum missing frames with differences for tracks, which decreases the number of identity switches of the same object and, thus, further improves the association accuracy. By using the proposed multi-stage association method, we develop a tracker called MSA-MOT for the 3D multi-object tracking task, alleviating the inherent matching problem in one-stage association. Extensive experiments are conducted on the challenging KITTI benchmark, and the results show that our tracker outperforms the previous state-of-the-art methods in terms of both accuracy and speed. Moreover, the ablation and exploration analysis results demonstrate the effectiveness of the proposed multi-stage association method.

## 1. Introduction

Multi-object tracking (MOT) is a key component of autonomous driving and robot navigation systems [1,2], which aims to obtain dynamic information about the environment by associating the objects in consecutive frames. Early multi-object tracking methods [3,4,5] are based on the images captured by camera and achieves promising results. However, the image information typically degrades in complex scenes (e.g., due to overexposure and insufficient light), leading to limited performance. In contrast, the point cloud data acquired by lidar include depth information of the scene [6], which shows robustness to complex conditions. Therefore, researchers are working on developing 3D multi-object tracking (3D MOT) frameworks [7,8] based on lidar sensors.

Existing 3D MOT methods can be mainly divided into single-modality-based [9,10] and multimodality-based [11,12,13] methods. Single-modality methods are generally based on lidar sensors for tracking. In contrast, multimodal approaches typically show better performance due to the use of complementary information. Multimodality methods [13,14,15] generally use 2D and 3D detectors to generate bounding boxes of objects, and then use a feature fusion module to fuse the different modal features provided by the feature extractor. Afterward, the objects are associated with candidate tracks based on similarity to perform tracking. However, most existing methods adopt one-stage association, failing to match the detections and tracklets with great accuracy. The one-stage association method filters out the low-score detections before input, and thus ignores the real objects contained in the low-score detections, which significantly affects the association accuracy. This method also does not re-track objects that are occluded for a long time, which leads to identity switching of the same object.

To solve the above problems, we propose a novel 3D multimodality tracker, namely, MSA-MOT, which is centered on a multi-stage association method consisting of a hierarchical matching module and a customized track management module. Specifically, to guide accurate association between detections and tracks, we propose a hierarchical matching module. First, 3D bounding boxes are projected to the image and matched with 2D bounding boxes to evaluate the detection reliability. Second, considering that most unreliable detections degrade the association accuracy, we only match the high-reliability detections with all candidate trajectories. Furthermore, the unmatched trajectories in the previous stage are matched with unreliable 3D detections, which increases the utilization of real objects in unreliable 3D detections. Finally, considering that some objects can be detected by camera but not by lidar (e.g., distant objects with low scores), we match the unreliable 2D detections with the remaining trajectories, reducing the number of missed detections of true objects at a distance. Based on this module, reliability information on tracklets is generated. To fully utilize this information to effectively manage the tracklets, we propose a customized track management module. Premature deletion of tracklets and tracking drift are the main reasons for object identity switching. Moreover, the tracks that are matched with 3D detections may not disappear in a short time. Therefore, we set larger maximum missing frames for reliable trajectories than for unreliable tracks, effectively reducing the number of identity switches in tracking.

**Figure 1:**
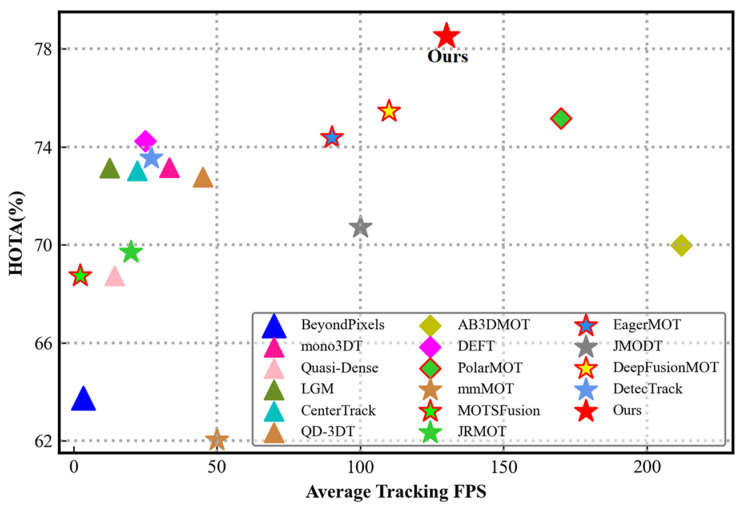
Comparison results of MSA-MOT with previous state-of-the-art methods. ▲ and ◆ represent camera-based and lidar-based methods, respectively. In addition, ★ represents multimodality-based methods. A red outline indicates that the method uses the same detector as our method. A higher value indicates better performance. The results show that MSA-MOT achieves accurate tracking with great speed. The results are presented in detail in Table 1.

To demonstrate the effectiveness and advancement of the proposed tracking method, we conduct a series of comparison experiments and ablation experiments on the challenging KITTI dataset [16]. As shown in Figure 1, the proposed MSA-MOT method achieves the highest Higher-Order Tracking Accuracy (HOTA), while running at a high speed of 130 frames per second (FPS), which verifies the effectiveness of our method.

To summarize, our main contributions are as follows:

- We propose a novel tracking method MSA-MOT for 3D MOT in complex scenes, in which we improve the association scheme by utilizing multi-stage association, and, thus, achieve precise tracking over a long period of time.
- In the multi-stage association method, the proposed hierarchical matching module successively associates the high- and low-reliability detections, alleviating the long-standing problem of incorrect association. In addition, a customized track management module is proposed for managing tracklets based on the information provided by the matching module, effectively addressing the severe identity switch in tracking.
- Extensive experiments are conducted on the challenging KITTI benchmark. The results show that MSA-MOT achieves state-of-the-art performance (78.52% on HOTA, 97.11% on sAMOTA, and 130 FPS), which demonstrates the effectiveness of our novel multi-stage association method.

The remainder of the paper is structured as follows: Section 2 discusses the related work. Section 3 details the structure of our proposed framework and analyzes each module. Section 4 presents the experiments and results analysis. Section 5 discusses the conclusions and future plans of this study.

## 2. Related Work

### 2.1. 2D MOT

Recent research on 2D MOT has mainly focused on two paradigms: tracking by detection and joint detection and tracking. The tracking by detection [17,18,19] paradigm is based on the detections provided by a detector and uses filters for state estimation such as Kalman [20]. Then, the detections and tracks are associated based on similarity to perform tracking. The joint detection and tracking [21,22] paradigm performs detection and tracking at the same time, and the same backbone network is used to extract features for both detection and tracking.

### 2.2. Single-Modality 3D MOT

With the rapid development of 2D MOT and 3D detectors [23,24,25], lidar-based 3D MOT has received great attention. For example, inspired by SORT [5], Weng et al. [7] developed a simple tracking method based on the Kalman filter, which uses the 3D intersection over union (3D IoU) and Hungarian algorithm [26] to perform tracking. Chiu et al. [27] proposed the replacement of the 3D IoU with the Mahalanobis distance [28], and initialized the covariance in the Kalman filtering process with the statistics of the training set. Zhai et al. [29] proposed a scene flow estimation network for obtaining implicit motion information, and then tracked the object of interest by an identity propagation strategy. Moreover, similar to CenterTrack [30], which is a 2D MOT tracker, Yin et al. [31] proposed representing each object by the center of its bounding box, and used speed estimation to achieve tracking without filtering. Wu et al. [32] proposed a tracker based on a data association scheme guided by prediction confidence. Kim et al. [33] explored the impact of geometric relationships between objects for 3D multi-object tracking based on graph neural networks. In addition, various methods [34,35,36] use other kinds of sensors to perform tracking.

In addition to the above works, some researchers are committed to using multiple cameras to estimate 3D bounding box information for 3D MOT. For example, Hu et al. [9] proposed the use of quasi-dense similarity learning to identify various objects with appearance characteristics, and then used a 3D bounding box depth sorting trial method for robust instance association. Marinello et al. [37] proposed the combination of triplet embedding and motion characterization for 3D object tracking.

### 2.3. Multimodality 3D MOT

Single-modality methods generally reduce the ability to obtain information in complicated situations, e.g., cameras are susceptible to interference from light intensity, and the point cloud data acquired by lidar are sparse when the object is at a distance. Therefore, multimodality methods for 3D MOT have recently attracted wide attention due to the sufficient information provided by sensors. For example, Zhang et al. [15] designed a sensor-agnostic multimodal framework for 3D MOT, which focuses on enabling joint optimization for the basic feature extractor of each mode and the adjacency estimator of the cross mode. Weng et al. [38] obtained both apparent and motion features from 2D and 3D space, and proposed a feature interaction mechanism based on a graph neural network. In addition, Zeng et al. [39] fused the point cloud with the corresponding image and mapped the lidar and camera features to a birds-eye-view using a 3D backbone. Chiu et al. [13] designed a probabilistic lidar and camera-based multi-object tracking system, and proposed an affinity combined with the Mahalanobis distance [28] and feature distance. Huang et al. [11] proposed the generation of bounding boxes and association scores from cameras and lidar data at the same time, and used a simple multiscale feature fusion scheme to estimate appearance affinities for tracking. Moreover, Gautam et al. [14] proposed the use of the deep learning model for correlation, in combination with the interacting multiple model (IMM) filter for state estimation. Koh et al. [40] proposed the use of the graph neural network (GNN) to associate objects based on spatiotemporal features, and combined rule-based edge pruning and attention-based edge control to improve the tracking performance. In addition, Nabati et al. [41] designed an end-to-end network for 3D MOT based on radar and camera sensor fusion, and they greedily used depth, velocity, and 2D displacement information to associate objects. However, the above methods typically associate all the detections and trajectories based on the similarity in one stage, failing to perform accurate matching between detections and tracklets.

Various methods have been developed for solving this problem. For example, Kim et al. [42] used 3D and 2D detectors to obtain multimodal detections. Then, they tracked the objects based on Kalman and a two-stage data association module. However, this method ignores the effects of low-score detections and track management on tracking performance, and, thus, cannot achieve long-term robust tracking. In addition, Wang et al. [43] proposed a deep association mechanism that establishes both 2D and 3D trajectories, and used the differences between the two types of tracks for tracking. However, this method fails to make full use of tracklet information. In this paper, we propose a better solution, namely, the multi-stage association approach MSA-MOT.

## 3. Methods

### 3.1. Overall Framework

**Figure 2:**
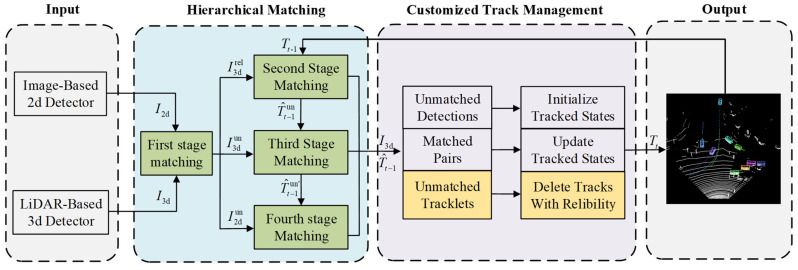
Proposed multi-stage association framework for the 3D multi-object tracking task. $I_{2d}$ and $I_{3d}$ indicate 2D and 3D detections, respectively. $I_{3d}^{\mathrm{rel}}$, $I_{3d}^{\mathrm{un}}$, and $I_{2d}^{\mathrm{un}}$ indicate the reliable 3D detections, unreliable 3D detections, and unmatched 2D detections, respectively, that are output from the first stage. In addition, $T_{t-1}$ indicates the candidate tracks of the previous frame. ${\hat{T}}_{t-1}$ denotes the trajectories of $T_{t-1}$ after prediction. ${\hat{T}}_{t-1}^{\mathrm{un}}$ and ${\hat{T}}_{t-1}^{{\mathrm{un}}^{\prime }}$ indicate unmatched tracks in the second and third stages, respectively. Moreover, $T_{t}$ denotes the output trajectories at the current frame.

**Figure 3:**
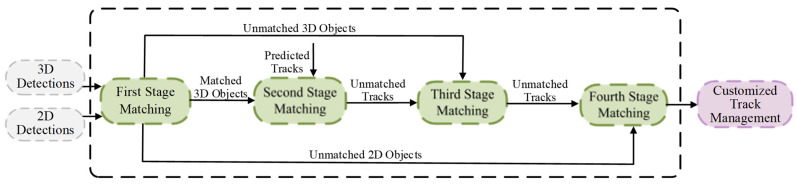
Framework of the hierarchical matching module, which contains four stages of matching.

We propose a multi-stage association tracker, MSA-MOT, for 3D multi-object tracking. The overall framework is shown in Figure 2, which includes a detection module, hierarchical matching strategy, and customized track management module. Specifically, in the detection module, to obtain multimodal bounding boxes, we use 2D and 3D detectors based on camera and lidar sensors, respectively. After that, the proposed hierarchical matching strategy is used to achieve accurate association, which includes four stages, as shown in Figure 3. First, the 3D bounding boxes are projected to the image dimension, and data matching with 2D detections is performed to judge the reliability of objects. Second, the reliable 3D detections are associated with candidate trajectories. Third, the unreliable 3D detections are associated with the unmatched trajectories. Finally, the remaining trajectories are projected to 2D and associated with the unreliable 2D detections. After these two modules, the customized track management module aims to initialize and update tracks, while focusing on efficiently managing the missing tracks. Specifically, based on the reliability of the tracks provided by the matching module, we set a larger maximum number of missing frames for reliable tracks than for unreliable tracks.

### 3.2. Hierarchical Matching Module

#### 3.2.1. First Stage of Matching

The first stage aims to prepare for the subsequent stages by determining the reliability of detections. For reliability judgment, a previously proposed method [44] is used to set a threshold based on the confidence provided by the detector. However, for this method, the score must be set manually, which is troublesome in practice. To solve this problem, we note that the objects detected by both 2D and 3D detectors are reliable. As a result, a strategy for reliability judgment based on multimodality information is proposed. Specifically, we obtain a set of 3D detections $I_{3d}$ and 2D detections $I_{2d}$ from 3D and 2D detectors, respectively, as expressed in Equations (1)–(3).

(1) $$I_{3d}=\left\{I_{3d}^{1},I_{3d}^{2},\cdots ,I_{3d}^{n}\right\}$$

(2) $$I_{3d}^{i}=\left\{x,y,z,\theta ,l,w,h\right\},i\in \left\{1,2,\cdots ,n\right\}$$

(3) $$I_{2d}=\left\{I_{2d}^{1},I_{2d}^{2},\cdots ,I_{2d}^{m}\right\}$$

where *n* and *m* denote the numbers of 3D detections and 2D detections, respectively; (*x*, *y*, *z*) denotes the center coordinates of the corresponding 3D objects; θ denotes the yaw angle; *l*, *w*, and *h* denote the length, width, and height, respectively, of the bounding box. For each 3D bounding box, we replace the polygon box with a precise rectangular box after projecting to 2D. The projected 2D detection set is represented as $I_{3d}^{proj}$, corresponding to $I_{3d}$. We obtain two sets of 2D detections ($I_{3d}^{\mathrm{proj}}$ and $I_{2d}$). Each bounding box in 2D is denoted by $B_{2d}$ as expressed in Equation (4).

(4) $$B_{2d}=\left(X_{1},X_{2},Y_{1},Y_{2}\right)$$

where (*X*_1_, *Y*_1_) and (*X*_2_, *Y*_2_) denote the upper-left and lower-right coordinates, respectively, of the bounding box. Then, we use the Euclidean distance based on (*X*_1_, *X*_2_, *Y*_1_, *Y*_2_) and the greedy algorithm [45] to associate the two sets of 2D detections, as expressed in Equation (5).

(5) $$Output=\mathrm{Greedily}\left(L2(I_{3d}^{\mathrm{proj}},I_{2d})\right)$$

where Greedily (·) denotes the greedy algorithm. L2 (·) denotes the Euclidean distance.

Finally, three sets of detections are output for subsequent stages: 3D objects that match with 2D detections, which are considered reliable and denoted $I_{3d}^{\mathrm{rel}}$; 3D objects that fail to match, which are referred to as unreliable detections and denoted $I_{3d}^{\mathrm{un}}$; unmatched 2D detections, which are denoted $I_{2d}^{\mathrm{un}}$.

#### 3.2.2. Second Stage of Matching

To reduce the impact of unreliable 3D detection and, thus, increase the association accuracy, we only associate the candidate trajectories with reliable 3D detections $I_{3d}^{\mathrm{rel}}$ in this stage. Specifically, we first use the constant-velocity motion model and Kalman filter to obtain the prediction state ${\hat{T}}_{t-1}$ of the previous trajectory set $T_{t-1}$. The prediction state is defined in Equation (6):

(6) $${\hat{T}}_{t}^{k}=\left\{\hat{x},\hat{y},\hat{z},\theta ,l,w,h,v_{x},v_{y},v_{z}\right\}$$

where *k* denotes one of the predicted trajectories, $\left(\hat{x},\hat{y},\hat{z}\right)$ denotes the predicted center coordinates, and $v_{x},v_{y\;},\;\mathrm{and}\;v_{z}$ denote the velocity of motion in 3D space. Then, to accurately calculate the similarity between detections and trajectories, we propose a new affinity metric that considers the coordinates and rotation angles of the objects, which is defined in Equation (7):

(7) $$A=L2(P_{\det},P_{\mathrm{pred}})\times (2-\cos(\alpha_{\det},\alpha_{\mathrm{pred}}))$$

where L2 denotes the Euclidean metric; $P_{\det},P_{\mathrm{pred}}$ denote the coordinates of the diagonal of the detection and the track, respectively; α denotes the yaw angle of the corresponding bounding box.

Then, we match the 3D detections with all the candidate tracks based on the proposed affinity and greedy algorithm. Finally, the unmatched trajectories ${\hat{T}}_{t-1}^{\mathrm{un}}$ are used as input for the next stage.

#### 3.2.3. Third Stage of Matching

In this stage, we focus on the detections $I_{3d}^{\mathrm{un}}$ output from the first stage. Because their existence is reasonable, we believe that the unreliable detections $I_{3d}^{\mathrm{un}}$ still include some real objects, such as heavily obscured objects that can be detected only by the 3D detector. Therefore, considering the components of $I_{3d}^{\mathrm{un}}$, we still use it to recover the real objects in unreliable detections. Specifically, we greedily match $I_{3d}^{\mathrm{un}}$ with ${\hat{T}}_{t-1}^{\mathrm{un}}$ based on the proposed affinity to further achieve accurate associations. Finally, the tracks that are unmatched again are named ${\hat{T}}_{t-1}^{{\mathrm{un}}^{\prime }}$.

#### 3.2.4. Fourth Stage of Matching

Finally, to obtain more accurate trajectories, we use the unmatched 2D detections $I_{2d}^{\mathrm{un}}$ provided by the first stage to recover the objects that only detected by the camera sensor. Specifically, ${\hat{T}}_{t-1}^{{\mathrm{un}}^{\prime }}$ failing several times to match in the third stage does not mean that all these tracklets have left the field of view. For example, for objects at long distances, because the point cloud data are sparse but the image information is relatively rich, it is likely that the 3D detector cannot detect them, but the 2D detector can. Therefore, to make full use of 2D information, we match the unmatched 2D detections $I_{2d}^{\mathrm{un}}$ with ${\hat{T}}_{t-1}^{{\mathrm{un}}^{\prime }}$ based on the Euclidean distance and greedy algorithm. Finally, the matching result is used as a new message for those tracks.

### 3.3. Customized Track Management Module

**Figure 4:**
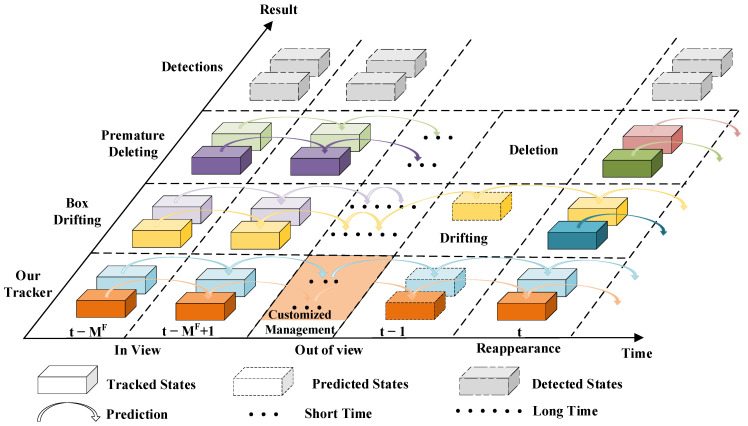
Illustration of existing problems in track management module. Different colored bounding boxes indicate different objects. If the candidate trajectory is deleted early, a new trajectory will be generated, resulting in identity switching of the object. In addition, if the trajectory is retained for too long, the trajectory will drift and, thus, generate an incorrect association.

The track management module aims to initialize, update, and delete the candidate tracks. For the track initialization, we set the minimum hitting frames commonly used in 3D MOT to determine whether a new trajectory needs to be initialized. Then, for the update process, we use the 3D detections that match the candidate 3D tracks to update the status of the corresponding trajectories based on Kalman filtering, and the updated tracks are denoted $T_{t}$, which is defined in Equation (8).

(8) $$T_{t}^{s}=\left\{\dot{x},\dot{y},\dot{z},\dot{\theta },\dot{l},\dot{w},\dot{h},{\dot{v}}_{x},{\dot{v}}_{y},{\dot{v}}_{z}\right\}$$

where *s* represents one of the updated tracks and the variables with “·” correspond to the updated value. For the trajectories that only match with $I_{2d}^{\mathrm{un}}$, considering that it is difficult to accurately transform 2D bounding boxes to the 3D coordinate system, we only use the prediction results obtained by Kalman filtering as the new states of the trajectories and add them into $T_{t}$. Finally, we delete candidate trajectories based on the reliability of the tracks to reduce the identity switching in the tracking process. We consider two factors that may lead to identity switching as shown in Figure 4: candidate trajectories are deleted prematurely, failing to be re-tracked later, and tracks drift due to predictions that are too long, which cannot be accurately matched again.

To alleviate these problems, we note that objects detected by 3D detectors may exist for a long time, while objects recognized only by camera-based detectors may leave from view more quickly. Therefore, we propose a new strategy based on the information provided by the matching module for reducing identity switches. Specifically, we regard trajectories that match with 3D detections as reliable and, thus, set a large maximum missing frames (M^F^) for them. A track is deleted when the number of unmatched consecutive frames of the track is higher than M^F^. In addition, for trajectories that only match 2D detections, we set M^F^ to smaller values to reduce interference with reliable trajectories, as defined in Equation (9). Experiments show that this strategy can effectively reduce the number of redundant tracks and improve the tracking accuracy.

(9) $$M^{F}=\left\{\begin{array}{l} H_{Fames},\;{\hat{T}}_{t-1}^{i}⊗I_{3d}^{m}\\ L_{Frames},\;{\hat{T}}_{t-1}^{i}⊗I_{2d}^{n} \end{array}\right.,\;{\hat{T}}_{t-1}^{i}\in {\hat{T}}^{}{}_{t-1}$$

where $H_{Frames},L_{Frames}$ represent high and low frames, respectively; ${\hat{T}}_{t-1}^{i}$ represents a candidate track; $I_{3d}^{m},I_{2d}^{n}$ denote one of the 3D and 2D detections; ⊗ denotes successful association.

## 4. Experiments

### 4.1. Dataset

We evaluate our method on the challenging KITTI [16] benchmark. KITTI provides both image and point cloud data, collected by a front camera and Velodyne HDL-64E lidar scanner, respectively. The dataset contains 21 training sequences and 29 testing sequences. We follow GNN3DMOT [38] to separate the training sequence. The sub validation set consists of 10 sequences, and the training set consists of 11 sequences. Because the KITTI dataset only provides ground truth labels for the training/validation split, all the ablation experiments in the paper are performed on the validation set.

### 4.2. Evaluation Metrics

To fully demonstrate the effectiveness of MSA-MOT, we evaluate both the 2D and 3D MOT performances. For 2D MOT, the tracking results are obtained by projecting the 3D bounding boxes to the image plane. We use widely used metrics [46,47] to evaluate the performance of 2D MOT, e.g., HOTA, Association Accuracy (AssA), Multi-Object Tracking Accuracy (MOTA), and Identity Switch (IDSW). For 3D MOT evaluation metrics, we use the scaled Accuracy Multi-Object Tracking Accuracy (sAMOTA) and the Averaged Multi-Object Tracking Accuracy (AMOTA) proposed in AB3DMOT [7]. sAMOTA is defined in Equation (10):

(10) $$\mathrm{sAMOTA}=\frac{1}{L}\sum_{r\in \left\{\frac{1}{L},\frac{2}{L},\cdots ,1\right\}}{\mathrm{sMOTA}}_{r}$$

(11) $${\mathrm{sMOTA}}_{r}=\max(0,1-\frac{{\mathrm{IDS}}_{r}+{\mathrm{FP}}_{r}+{\mathrm{FN}}_{r}-(1-r)\times {\mathrm{GT}}_{r}}{r\times {\mathrm{GT}}_{r}})$$

where *r* is the recall value (confidence threshold), *L* is the number of recall values, and IDS*_r_*, FP*_r_*, FN*_r_*, and GT*_r_* represent the numbers of identity switches, false positives, false negatives, and ground truths, respectively.

### 4.3. Implementation Details

All the experiments are implemented using Python 3.7 with an Intel Core i5 11400F 2.6 GHz CPU and 16 GB of RAM. We use PointGNN [48] as a 3D detector to predict 3D bounding boxes and use RRC [49] for 2D images. The affinity thresholds for 2D and 3D are set to 25 and 7, respectively. For tracklets with high reliability, the maximum number of missing frames is 11, and the maximum number of missing frames for the remaining tracklets is set to 3. In the 3D MOT performance evaluation, we set IoU = 0.25 to conduct a fair comparison with other works.

### 4.4. Comparison with the State-of-the-Art Methods

#### 4.4.1. Quantitative Comparison

2D MOT: Table 1 shows the comparison results of the proposed method for the car class of the KITTI MOT testing set. To demonstrate the effectiveness of our method, we compare MSA-MOT with current state-of-the-art methods (including single modality-based and multimodality-based methods). The results show that the proposed method achieves the highest HOTA (78.52%) and AssA (82.56%) among the compared methods. It also shows superior performance in terms of the remaining metrics (MOTA, IDSW, and FPS), reaching 88.01%, 91, and 130, respectively. Specifically, MSA-MOT outperforms DeepFusionMOT, which uses the same 2D detector as our method, by significant margins, namely, 3.06% and 3.37% for HOTA and MOTA, respectively. In addition, compared to EagerMOT with the same 2D and 3D detectors, our method realizes 4.13% and 8.4% improvements in HOTA and AssA, respectively, due to the effectiveness of our multi-stage association. Notably, our method achieves significant improvements in the AssA metric, which indicates the accuracy of data association. This is mainly attributed to the proposed hierarchical module. In addition, due to the customized track management module, the IDSW value is much smaller than those of most state-of-the-art methods. Moreover, our method balances accuracy and speed.

3D MOT: To further evaluate our method, we compare the performance of 3D MOT on the KITTI validation set for the car class. As shown in Table 2, MSA-MOT outperforms the previous state-of-the-art methods, achieving the highest sAMOTA (97.11%), AMOTA (50.10%), and MOTA (96.83%). Specifically, MSA-MOT outperforms the lidar-based PolarTrack, which uses the same 3D detector, by 2.79% and 2.9% in terms of sAMOTA and MOTA, respectively. In addition, compared to DeepFusionMOT, which uses the same 2D detector, the proposed method achieves remarkable improvements (approximately 5%) in the key metrics sAMOTA, AMOTA, and MOTA. Moreover, compared to EagerMOT with the same 2D and 3D detectors, sAMOTA and AMOTA are improved by 2.17% and 1.26%, respectively. Moreover, our method demonstrates significant superiority compared to multimodality methods such as mmMOT, GNN3DMOT, and DetecTrack. All performance advantages are due to the proposed multi-stage association method.

#### 4.4.2. Qualitative Comparison

**Figure 5:**
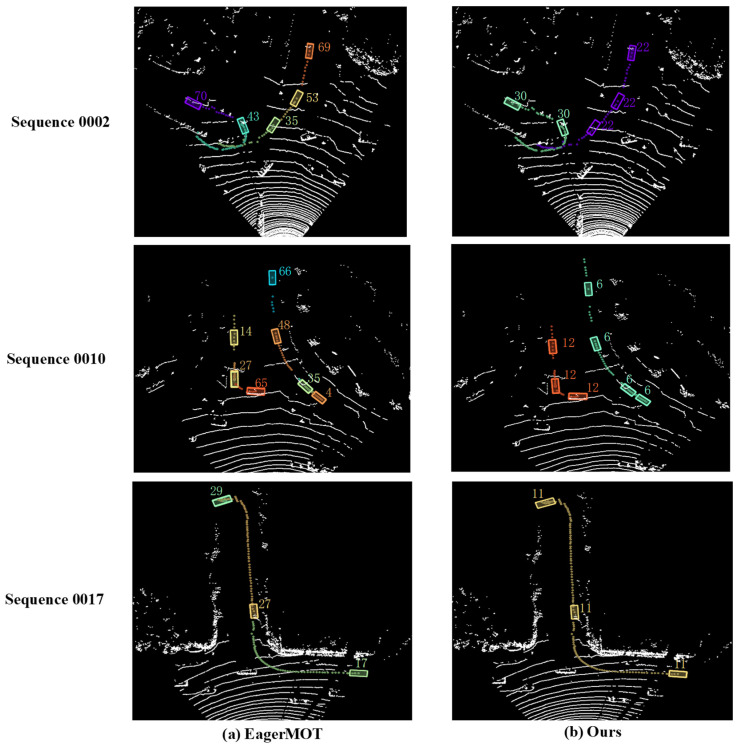
Visualization results of EagerMOT and our proposed method in the lidar view for three sequences. We only visualize the trajectories of one or two objects for each sequence to make an obvious comparison. Each colored point represents the center of the corresponding bounding box, and the dashed line formed by the points indicates the historical or predicted trajectory of the object. Different-colored bounding boxes indicate different IDs of the object in the sequence. The ID of the object is annotated next to each bounding box.

To visually demonstrate the superiority of the proposed method, we perform a qualitative comparison on the KITTI dataset. EagerMOT is a classic method for 3D MOT and uses the same 2D and 3D detectors as our method, so we choose it as the method for comparison. Figure 5 shows the visualization results of EagerMOT and MSA-MOT in the lidar coordinate system. The left and right columns represent the visualizations corresponding to EagerMOT and our method, respectively. Specifically, in sequence 0002, the object with ID 43 in Figure 5a changes its ID to 70 while turning around. However, our method performs accurate tracking all the time, which demonstrates that MSA-MOT can robustly track dynamically changing objects. In sequence 0010, some colored points are far apart, which means that the objects disappear from the view for a long time. In this case, because the proposed track management module can effectively retrack the occluded objects, MSA-MOT still tracks the objects robustly, whereas with EagerMOT, the IDs of objects change multiple times (the ID of a car changes from 4 to 35, 48, and 66). In addition, it is challenging to track objects accurately when they are at a distance. However, in sequence 0017, due to the proposed hierarchical matching module reducing the loss of real objects caused by the low-scoring detections, our method accurately tracks the car with ID 11 while it is driving away.

**Figure 6:**
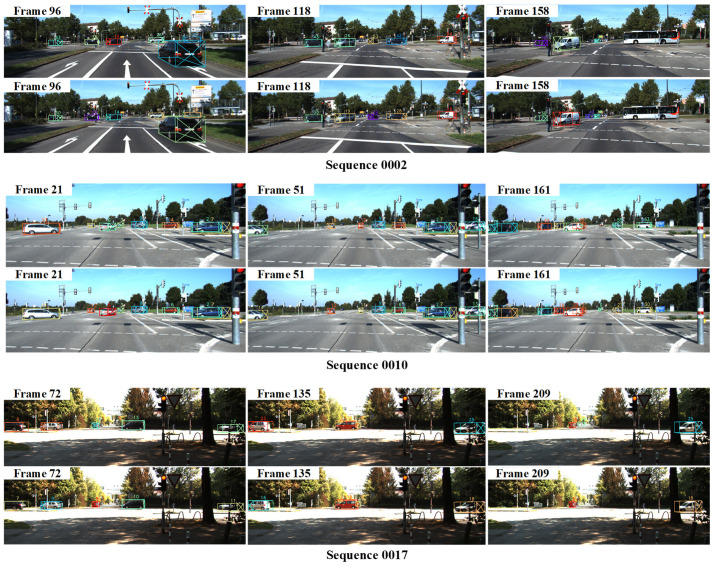
Visualization results of sequences 0002, 0010, and 0017 in the camera view. The images presented above are obtained by EagerMOT for each sequence, and the images below represent the results obtained by our method. Different objects are represented by different colors. This shows that our method can perform accurate tracking for a long time.

To further demonstrate the effectiveness of our method, we choose three frames for the above sequences in the image dimension, as shown in Figure 6. The upper and lower parts of each sequence represent the visualization results of EagerMOT and MSA-MOT, respectively. Specifically, in sequence 0002, due to the change in motion stage and long-term occlusion, the vehicle with ID 43 obtained by EagerMOT changes its ID in the 158th frame. However, our method achieves accurate tracking of the car. In addition, in sequence 0010, due to the severe obscuration by other objects, the car with ID 14 is incorrectly tracked many times by EagerMOT. However, our method always performs precise tracking. In addition, in sequence 0017, the vehicle with ID 11 drives far away, and its surrounding light is bright. It is difficult to track the object accurately under these conditions, but our tracker still achieves this. The results show that our method can achieve superior tracking performance under challenging conditions, such as long-term occlusion, sudden changes in the motion state, and objects at a distance.

### 4.5. Ablation Experiments

#### 4.5.1. Component-Wise Analysis

To demonstrate the effectiveness of the proposed components, i.e., the hierarchical matching module and customized track management module, and investigate their contributions to the tracking performance, we conduct an ablation study on the KITTI validation set. For a fair analysis, we still choose EagerMOT (using the same 2D and 3D detectors) as the comparison method, as shown in Table 3. Due to the utilization of positive objects with low reliability in our hierarchical matching module, a 0.99% performance improvement in HOTA is realized, along with a 2.58% improvement in AssA. In addition, by combining all the modules, the performance is further enhanced, which indicates that the customized track management approach can improve the association accuracy and reduce the number of identity switches.

#### 4.5.2. Hierarchical Matching Module

The affinity used to evaluate the similarity between the detections and trajectories has a crucial influence on the tracking performance. To further demonstrate the effectiveness of the proposed hierarchical matching strategy, we perform an ablation analysis with EagerMOT under the commonly used 3D intersection over union (3D-IoU) [7] and 3D generalized intersection over union (3D GIoU) [55] metrics, as shown in Table 4. The results show that our method outperforms EagerMOT with the same detector in terms of different metrics. Moreover, the affinity metric proposed in this paper outperforms the commonly used affinity metric.

#### 4.5.3. Track Management Module

In addition, we ablate the maximum number of missing frames on the KITTI validation set for the car class, as shown in Table 5. When the maximum missing frames is set to 5, the module shows suboptimal performance, which is caused by tracks being deleted prematurely. A larger value results in many redundant candidate tracklets; thus, the performance shows a decreasing trend. Notably, the best performance is achieved when the maximum missing frames is set to 11. If not specified, this value is set to 11 for all experiments in this paper.

### 4.6. Exploration Analysis

To further demonstrate the superiority of our method, we conduct an exploratory analysis. As the tracking performance of AB3DMOT differs among IoU threshold values (used to calculate the similarity between the tracking results and ground truths), we set different IoU threshold (IoU_thres_) values, as in AB3DMOT, to verify the robustness of our method, as shown in Table 6. Compared to the two classic methods, our method almost ranks first in terms of various metrics. Moreover, although accurate tracking of pedestrians is generally challenging due to the dense bounding boxes, our method still obtains the best performance in terms of the two keys metrics (sAMOTA and AMOTA).

In addition, because the performance of the tracker is partly dependent on the detector, we compare the performance of our method under different 3D detectors. As shown in Table 7, we use three 3D detectors (Point-GNN [48], PointRCNN [24], and PV-RCNN [56]) that are widely applied in 3D MOT tasks. Experimental results show that our method still achieves superior performance with different detectors, proving that MSA-MOT has wide applicability and can be combined with various 3D detectors for robust tracking.

## 5. Conclusions

In this paper, we propose a novel multi-stage association framework for 3D multimodality multi-object tracking. In this framework, a hierarchical matching module is proposed to improve the utilization of true objects by matching detections and tracks in sequence, which achieves accurate association between detections and tracks. Then, based on the track reliability output from the matching module, the proposed customized track management sets larger maximum missing frames for reliable trajectories than for unreliable tracks, which further improves the association accuracy. Comprehensive experiments are conducted on the challenging KITTI benchmark. Our method achieves state-of-the-art performance among the competitive approaches. Abundant ablation experiments further demonstrate the effectiveness of the proposed modules.

MSA-MOT follows the commonly used constant-velocity motion model to predict the state of the object, where it is difficult to deal with the complex change of state. Therefore, a more accurate state estimation method is worth exploring in the future work.

## Figures and Tables

**Table 1:** Comparison results on the KITTI tracking testing benchmark for the car class. A higher value in the table indicates better performance without IDSW. Bold font indicates the best performance among all the compared methods, and the values that correspond to the second-best performance are underlined. “#” indicates that the method uses the same 3D/2D detector as our method.

Method	Publication	Input	HOTA (%)	AssA (%)	MOTA (%)	IDSW	FPS
BeyondPixels [50]	ICRA’18	2D + 3D	63.75	56.40	82.68	934	3.3
mmMOT [15]	ICCV’19	2D + 3D	62.05	54.02	83.23	733	50
mono3DT [51]	ICCV’19	2D	73.16	74.18	84.28	379	33.3
AB3DMOT [7]	IROS’20	3D	69.99	69.33	83.61	113	**212**
MOTSFusion [52] #	RA-L’20	2D + 3D	68.74	66.16	84.24	415	2.3
JRMOT [12]	IROS’20	2D + 3D	69.61	66.89	76.95	271	20
CenterTrack [30]	ECCV’20	2D	73.02	71.20	**88.83**	254	22.2
Quasi-Dense [53]	CVPR’21	2D	68.45	65.49	84.93	313	14.3
LGM [54]	ICCV’21	2D	73.14	72.31	87.60	448	12.5
JMODT [11]	IROS’21	2D + 3D	70.73	68.76	85.35	350	100
EagerMOT [42] #	ICRA’21	2D + 3D	74.39	74.16	87.82	239	90
TripletTrack [37]	CVPRW’22	2D	73.58	74.66	84.32	322	-
QD-3DT [9]	TPAMI’22	2D	72.77	72.19	85.94	206	45
PolarMOT [33] #	ECCV’22	3D	75.16	76.95	85.0	462	170
DeepFusionMOT [43]#	RA-L’22	2D + 3D	75.46	80.06	84.64	**84**	110
DetecTrack [40]	AAAI’22	2D + 3D	73.54	75.25	85.52	-	27
MSA-MOT	Ours	2D + 3D	**78.52**	**82.56**	88.01	91	130

**Table 2:** Comparison results of 3D MOT on the KITTI tracking validation set for the car class. The bold font indicates the best performance among all the compared methods.

Method	Publication	Modality	sAMOTA (%)	AMOTA (%)	MOTA (%)	IDS
FANTrack	IV’19	3D + 2D	82.97	40.03	74.30	35
mmMOT	ICCV’19	3D + 2D	70.61	33.08	74.07	10
AB3DMOT	IROS’20	3D	93.28	45.43	86.24	**0**
GNN3DMOT	CVPR’20	3D + 2D	93.68	45.27	84.70	**0**
EagerMOT#	ICRA’20	3D + 2D	94.94	48.84	96.61	2
PC-TCNN	IJCAI’21	3D	95.44	47.64	-	1
PolarTrack#	ECCV’22	3D	94.32	-	93.93	31
DetecTrack	AAAI’22	3D + 2D	96.49	48.87	91.46	-
DeepFusionMOT#	RA-L’22	3D + 2D	91.80	44.62	91.30	1
MSA-MOT	Ours	3D + 2D	**97.11**	**50.10**	**96.83**	**0**

**Table 3:** Ablation study results on the validation set for the car class, using the hierarchical matching strategy (MSM) and customized track management module (CTM). Because the proposed CTM relies on the results of the matching module, it is not ablated separately. Bold font indicates the best performance.

	MSM	CTM	HOTA (%)	DetA (%)	AssA (%)	MOTA (%)	IDSW
EagerMOT			78.04	76.80	79.51	87.25	91
Ours	√		79.03	77.39	80.90	88.23	66
	√	√	**79.73**	**77.50**	**82.09**	**88.49**	**46**

**Table 4:** Ablation study results for hierarchical matching strategy on the KITTI validation set for the car class. Eager denotes EagerMOT using the same 2D and 3D detectors. Bold font indicates the best performance.

Affinity	HOTA (%)		AssA (%)		MOTA (%)		IDSW	
	Ours	Eager	Ours	Eager	Ours	Eager	Ours	Eager
3D-IoU	78.83	77.16	80.34	77.76	88.00	86.67	206	234
3D-GIoU	79.41	77.82	81.50	79.08	88.45	87.09	95	126
Ours	**79.73**	78.12	**82.09**	79.67	**88.49**	87.27	**46**	79

**Table 5:** Ablation study for the maximum number of missing frames on the KITTI validation set for the car class. Bold font indicates the best performance.

Frames	HOTA (%)	AssA (%)	MOTA (%)	IDSW
5	79.06	80.89	88.40	56
8	79.24	81.23	88.48	50
11	**79.73**	**82.09**	**88.49**	**46**
14	79.65	82.06	88.48	46
17	79.64	82.02	88.45	46

**Table 6:** Comparison of 3D MOT performance on the KITTI validation set with different IoU thresholds for the car and pedestrian classes. Bold font indicates the best performance.

Method	Criteria	sAMOTA (%)		AMOTA (%)		MOTA (%)	
		Car	Pedestrian	Car	Pedestrian	Car	Pedestrian
AB3DMOT	IoU_thres_ = 0.25	93.28	75.85	45.43	31.04	86.24	70.90
	IoU_thres_ = 0.5	90.38	70.95	42.79	27.31	84.02	65.06
	IoU_thres_ = 0.7	69.81	-	27.26	-	57.06	-
EagerMOT	IoU_thres_ = 0.25	94.94	92.95	48.84	45.96	96.61	93.14
	IoU_thres_ = 0.5	95.42	90.57	48.93	43.79	94.67	90.66
	IoU_thres_ = 0.7	85.13	64.49	39.06	21.91	84.04	**64.67**
MSA-MOT	IoU_thres_ = 0.25	**97.11**	**93.61**	**50.10**	**46.31**	**96.83**	**94.63**
	IoU_thres_ = 0.5	**96.99**	**91.92**	**49.85**	**44.01**	**94.84**	**91.29**
	IoU_thres_ = 0.7	**86.85**	**66.77**	**39.90**	**23.58**	**84.16**	64.60

**Table 7:** Ablation results on the 3D detector for the car and pedestrian classes. Bold font indicates the best performance.

3D Detector	Car			Bicycle		
	SAMOTA (%)	MOTA (%)	IDs	SAMOTA (%)	MOTA (%)	IDs
Point-GNN	**97.21**	**96.68**	**0**	94.11	94.47	17
PointRCNN	97.18	95.44	0	81.37	81.95	**2**
PV-RCNN	94.56	95.54	0	**94.63**	**95.18**	6

## Data Availability

The publicly available dataset used in this paper can be found here: http://www.cvlibs.net/datasets/kitti/eval_tracking.php (accessed on 10 September 2022).
